# Differences in Medication Adherence between Living and Deceased Donor Kidney Transplant Patients

**Published:** 2014-02-01

**Authors:** K. Denhaerynck, G. Schmid-Mohler, A. Kiss, J. Steiger, R. P. Wüthrich, A. Bock, S. De Geest

**Affiliations:** 1 *Institute of Nursing Science, University of Basel, Switzerland*; 2 *Division of Nephrology, University Hospital Zürich, Switzerland*; 3 *Centre of Clinical Nursing Science, University Hospital Zürich, Switzerland*; 4 *Division of Psychosomatic Medicine, University Hospital Basel, Switzerland*; 5 *Transplantation Immunology and Nephrology, University Hospital Basel, Switzerland*; 6 *Division of Nephrology, Kantonsspital, Aarau, Switzerland*; 7 *Center of Health Services and Nursing Research, K.U. Leuven, Leuven, Belgium*

**Keywords:** Living related transplantation, Immunosuppressant adherence, Kidney transplantation, Donor, Graft

## Abstract

Background: Literature review suggests that adherence to immunosuppressive drugs may be lower in recipients of living than of deceased donor kidney grafts, possibly because of profile differences.

Objective: To compare the level of immunosuppressive adherence levels between patients with deceased and living (-related; -unrelated) donor grafts in Switzerland.

Methods: Using data from two similar cross-sectional studies at two transplant centers in Switzerland, the level of adherence between the two groups was compared. Medication adherence was assessed by self-report or electronic monitoring. Possible explanatory factors included age, beliefs regarding immunosuppressive drugs, depressive symptomatology, pre-emptive transplantation, and the number of transplants received, were also considered. Data were analyzed using logistic regression analysis.

Results: Unadjusted non-adherence odds were 2 to 3 times higher in living-related than deceased donor transplantation (ORs: 2.09-3.05; p<0.05). Adjustment for confounders showed that these differences were associated most with the younger age of living-related subjects and the belief that immunosuppressive drugs are less important for living-related donations.

Conclusion: There is a lower immunosuppressive adherence in recipients of living-related donor kidneys, possibly owing to differences in patient profile (*ie*, health beliefs regarding their immunosuppressive needs), knowledge of which may enhance adherence if addressed.

## INTRODUCTION

Transplantation is a cost-effective treatment for patients with end-stage renal disease [1, 2], providing them with improved quality of life and good long-term survival prospects. Survival rates are especially favorable for those who receive kidneys from living donors [3, 4], even under conditions of suboptimal tissue matching [5, 6], and where levels of non-adherence to immunosuppressive medication are comparatively high [7-11]. Higher non-adherence levels observed in recipients of living donor grafts have previously been attributed to a sense of relative invulnerability resulting from recipients’ younger age and to the possibility that those receiving grafts from relatives believe that their higher histocompatibility protects them adequately against rejection [9]. Also, a strong sense of obligation towards the donor overburdening these patients [12], or limited experience with self-management of chronic illness (*eg*, less experience with dialysis), implying fewer accumulated skills necessary to master a chronic condition such as living with a transplant [10, 13], are suggested as explanations for this group’s higher non-adherence levels. Because of the shortage of empirical data regarding the existence and/or extent of the supposed non-adherence differences, the primary objective of this study was to examine whether adherence differences could be detected between recipients of different types of kidney grafts. Should such differences be confirmed, our second goal was to examine a selection of variables acting as possible confounders. 

## MATERIALS AND METHODS

Two cross-sectional adherence studies in kidney transplant recipients were performed at the transplant centers of Zürich and Basel (Switzerland), approved by the responsible ethical committees (Ethikkommission Zürich 07/12.9.207; Ethikkommssion beider Basel 55/00; Überregionale Ethikkommision für klinische Forschung PV124/00-SNF). The studies differed from each other in their measurement of the number of variables reflecting possible reasons for adherence differences, but were similar in their measurement of the key variables, *ie*, adherence and graft type, the latter of which added to the literature in that, alongside deceased donation, it subdivided the living donation group into “living-related” and “living-unrelated.”

The Zürich study 

Design, Sample and Setting

The first source of data was a study conducted at the nephrology outpatient clinic at University Hospital Zürich [14], which is a site of about 85 renal transplants per year [15]. Patients were recruited consecutively during their follow-up visits. To qualify for inclusion, they had to be adult kidney transplant recipients one to five years after their first transplant, managing their medication independently, and able to write in German. Patients who lacked mental acuity were excluded. 

Procedure

Clinical variables were retrieved from participants’ medical files, including graft donor type (deceased, living-related, living-unrelated) and pre-emptive transplantation status (yes/no). Demographic and adherence data were assessed by interview. The item “how often have you not taken immunosuppressive medication in the past four weeks?” was selected as a measure of “taking adherence” from the 4-item Basel Assessment of Adherence Scale for Immunosuppressives (BAAS-IS) [16, 17]. 

The Basel study 

Design, Sample and Setting

The second source of data was a study conducted at the nephrology outpatient clinic of the University Hospital Basel [10], a similarly sized center conducting approximately 70 transplants annually [15]. The study’s convenience sample consisted of adult patients who were German or French speaking, literate, at least one year post-renal transplant, independently managing their immunosuppressive regimens, able to respond adequately to the researchers’ questions, and/or able to fill out the questionnaires.

Procedure

Patients were recruited during their annual check-up visits to the hospital, when demographic and adherence data were collected. Clinical variables retrieved from the patients’ medical files included graft donor type (deceased, living-related and living-unrelated), pre-emptive transplantation status (yes/no), number of HLA-mismatches, and number of transplants received. Depressive symptomatology was measured using the 21-item, 4-point Beck Depression Inventory [18, 19]. Patient beliefs with regard to immunosuppressive medications were also measured via a single-item measuring the respondent’s belief that an individual who receives a living donor kidney does not need as much immunosuppressive medication as a person who receives a kidney from a deceased donor. This was assessed on a 5-point Likert scale ranging from complete disagreement (‘1’) to complete agreement (‘5’). Non-adherence was assessed identically to the Zürich study [20].

A subsample of patients agreed to electronic adherence monitoring by means of a MEMS^®^-V TrackCap system (Aardex, Ltd.) [21]. The MEMS^®^ uses a medication bottle fitted with a cap containing a chip registering the time and date of every opening. For this analysis, taking adherence was defined as the ratio of registered openings to the number of prescribed doses [22]. For analytical reasons, the resulting data were transformed into three ordinal categories analogous to those used for self-reported non-adherence. 

Data analysis 

The two studies were analyzed separately. Data were not pooled to allow separate corroboration of our main hypothesis in the two different settings. Moreover, the Zürich study did not have the extended set of confounding factors. Variables were described using percentages, means and standard deviations. Simple comparisons of demographic and clinical variables between graft types were performed using χ^2^ and Mann-Whitney U tests. Testing for differences in adherence levels between graft types was done by ordinal logistic regression analysis [23], which calculated the probability that each patient was adherent. Secondary hypotheses were tested by adding the confounding variables of age, the belief that an individual who receives a living donor kidney does not need as much immunosuppressive medication as a person who receives one from a deceased donor, depressive symptomatology, number of pre-emptive transplantations, and number of transplants to the model. Analyses were performed using SAS version 9.1.3 (SAS Institute, NC, Cary).

## RESULTS

Sample characteristics 

Sample characteristics are outlined in Table 1. The Zürich study included 114 kidney transplant patients (74% of them eligible). The Basel study included 348 patients (86% of them eligible), of whom 248 had been assessed using electronic monitoring (60% of them eligible). In the Zürich study, half (51%) of the patients had received kidneys from living donors. In the Basel study, this figure was slightly lower (41%). Living-related and -unrelated grafts were represented equally in the Zürich study, whereas living-related donation was more prevalent in the Basel study (70% of living donations). In both studies, the mean age was 53 years; few were young adults (only three in each study were between 18 and 25 years).

**Table 1:** Demographic and clinical characteristics of the sample. Numbers are frequency (%) or mean±SD

**Variable**	**Values**	**Zürich study (n=114)**	**Basel study (n=348)**
Gender	Male	74 (64%)	207 (58%)
Living alone	No	15 (13%)	83 (23%)
Education	Higher education	24 (21%)	99 (27%)
Nationality	Swiss	87 (76%)	291 (81%)
Graft type	Deceased	56 (49%)	210 (59%)
	Living-related	28 (25%)	102 (29%)
	Living-unrelated	30 (26%)	43 (12%)
Immunosuppressive	Cyclosporine	47 (41%)	228 (64%)
drugs	Tacrolimus	55 (48%)	64 (18%)
	Sirolimus	11 (9%)	25 (7%)
	Azathioprine	13 (11%)	115 (32%)
	Corticosteroids	40 (35%)	93 (26%)
	Mycophenolate Mofetil	100 (87%)	170 (47%)
Mean±SD age		53.6119	52.913.5
Self-reported adherence	Adherent	96 (84.2%)	308 (88.3%)
	Non-adherent once a month	11 (9.7%)	28 (8.0%)
	Non-adherent ≥every 2 weeks	7 (6.1%)	13 (3.7%)
Mean±SD taking adherence (EM*)		—	97.3±8.9

* EM: Electronic monitoring

Table 2 shows that graft types did not differ with respect to the number of transplants or the levels of depressive symptoms. However, living-related transplant patients were younger, had a higher proportion of pre-emptive transplantations, showed fewer mismatches and had a stronger belief that an individual who receives a living donor kidney requires less immunosuppressive medication than one who receives a deceased-donor graft.

**Table 2:** Distribution of confounding factors

**Variable**	**Graft type**	**Zürich study** * **(n=114)**		**Basel study** * **(n=348)**	
Age (meanSD)	Deceased	56.410.6	p <.0001	56.713.6	p<0.001
	Living-related	45.814.4		44.811.0	
	Living-unrelated	55.48.0		54.39.5	
Pre-emptive transplantation† (frequency, %)	Deceased	2 (8%)	p<0.001	3 (8%)	p<0.001
	Living-related	13 (50%)		21 (54%)	
	Living-unrelated	11 (42%)		15 (39%)	
Health belief “with a living donor kidney, fewer immunosuppressives are needed as with a deceased donor kidney” (meanSD)	Deceased	—		2.131.19	p<0.001
	Living-related	—		2.721.44	
	Living-unrelated	—		1.931.27	
Number of transplants (meanSD)‡	Deceased	—		1.150.41	p=0.79
	Living-related	—		1.160.46	
	Living-unrelated	—		1.090.29	
Number of mismatches (meanSD)	Deceased	—		4.751.30	p<0.001
	Living-related	—		2.961.91	
	Living-unrelated	—		5.050.84	
Depressive symptomatology (mean std)	Deceased	—		0.360.32	p=0.35
	Living-related	—		0.360.33	
	Living-unrelated	—		0.290.26	

* χ² and Mann-Whitney U tests

† Recipients of transplants from a deceased donor who did not receive dialysis prior to transplantation were coded as pre-emptive

‡ Graft type of last transplantation reported

Differences in non-adherence among graft type groups 

In both studies living-unrelated graft recipients had adherence levels similar to those of deceased-donor recipients (Table 3). Similarly, self-reported non-adherence was significantly higher in recipients of living-related than in deceased grafts in both the Zürich study (OR=3.14; 95% CI: 1.01–9.82) and the Basel study (OR=2.09; 95% CI: 1.04–4.17). The same was true for electronically measured non-adherence (OR=2.12; 95% CI: 1.13–3.98). The extent to which confounding factors explain these latter adherence differences can be inferred by comparing the unadjusted ORs of Table 3 with their adjusted counterparts in Table 4. Controlling for the confounders lowered estimated self-reported non-adherence differences between living-related and deceased transplantation from unadjusted significant ORs between 2 and 3, to adjusted insignificant values of 1.08 in the Zürich study (95% CI: 0.25–4.60; p=0.92), 1.26 for the Basel study self-report (95% CI: 0.48–3.29; p=0.63), and 1.98 for the Basel study electronic monitoring (95% CI: 0.92–4.26; p=0.08). 

**Table 3:** Unadjusted logistic regression model

**Variable**	**Graft type**	**OR (95% CI)**	**R²**
Zürich study (SR*)	Deceased *vs* living-related	3.14 (1.01–9.82)	6.5%
	Deceased *vs* living-unrelated	0.80 (0.19–3.34)	
	Unrelated *vs* related	3.93 (0.92–16.7)	
Basel study (EM†)	Deceased *vs* living-related	2.12 (1.13–3.98)	3.0%
	Deceased *vs* living-unrelated	1.39 (0.57–3.39)	
	Unrelated *vs* related	1.53 (0.60–3.93)	
Basel study (SR)	Deceased *vs* living-related	2.09 (1.04–4.17)	2.2%
	Deceased *vs* living-unrelated	1.02 (0.33–3.16)	
	Unrelated *vs* related	2.05 (0.65–6.46)	

The coefficient of determination (R²) was calculated by the method presented by Nagelkerke, *et al*. [28].

* SR: Self-reported;

† EM: Electronic monitoring

**Table 4:** Adjusted logistic regression model

**Analysis**	**Variable**	**OR (95% CI)**	**R²**
Zürich study (SR*)	Graft type		
	Deceased *vs* living-related^†^	1.08 (0.25–4.60)	15.3%
	Deceased *vs* living-unrelated	0.48 (0.09–2.44)	
	Unrelated *vs* related	2.24 (0.47–10.7)	
	Controlled for		
	Age	1.06 (1.01–1.11)	
	Pre-emptive transplantation	0.30 (0.08–1.13)	
Basel study (EM‡)	Graft type		
	Deceased *vs* living-related^†^	1.98 (0.92–4.26)	9.6%
	Deceased *vs* living-unrelated	1.76 (0.65–4.77)	
	Unrelated *vs* related	1.12 (0.38–3.28)	
	Controlled for		
	Age	1.02 (1.00–1.05)	
	Health belief	0.85 (0.67–1.06)	
	Number of mismatches^§^	0.92 (0.75–1.14)	
	Pre-emptive transplantation	1.29 (0.44–3.74)	
	Number of transplantations	0.57 (0.32–1.04)	
	Depressive symptoms	0.53 (0.22–1.21)	
Basel study (SR)	Graft type		
	Deceased *vs* living-related^†^	1.26 (0.48–3.29)	8.1%
	Deceased *vs* living-unrelated	1.06 (0.31–3.65)	
	Unrelated *vs* related	1.18 (0.32–4.43)	
	Controlled for…		
	Age	1.02 (0.99–1.05)	
	Health belief	0.71 (0.55–0.92)	
	Number of mismatches	1.04 (0.83–1.29)	
	Pre-emptive transplantation	0.80 (0.28–2.31)	
	Number of transplantations	1.00 (0.41–2.43)	
	Depressive symptoms	1.28 (0.38–4.26)	

* SR: Self-reported

† Estimate to be compared with in Table 5

‡ EM: Electronic monitoring

§ Matching was added as a variable to be able to estimate the effect of the belief in the need for less immunosuppressives regardless of the actual histocompatibility

A single confounder’s contribution to explaining adherence differences between deceased and living-related transplants was inferred by omitting one explanatory variable at a time from the model (Table 5), revealing that estimated ORs were affected most by omitting age and the belief that an individual who receives a living donor kidney needs less immunosuppressive medication than the recipient of a deceased-donor graft. The effect of pre-emptive transplantation on the ORs differed between the studies.

**Table 5:** Contribution of single variables to the logistic regression model. This Table provides an estimation of the relative contribution of explanatory variables to explaining adherence differences between deceased vs living-related graft recipients. The odds ratio represents the contrast between deceased vs living-related graft recipients and, which can be compared to Table 4

	**Variable omitted from the regression model**	**Parameters for contrasting deceased vs living-related graft recipients **	
		Adjusted OR (95% CI)	R²
Zürich study (SR*)	Age	2.11 (0.57–7.84)	8.7%
	Pre-emptive transplantation	2.01 (0.57–6.97)	11.5%
Basel study (EM†)	Age	2.38 (1.14–5.00)	8.1%
	Health belief	2.02 (0.94–4.31)	8.2%
	Number of mismatches	1.70 (0.83–3.45)	9.3%
	Pre-emptive transplantation	1.91 (0.90–4.05)	9.6%
	Number of transplantations	1.91 (0.88–4.12)	7.9%
	Depressive symptoms	1.94 (0.91–4.14)	7.6%
Basel study (SR)	Age	1.65 (0.67–4.02)	6.9%
	Health belief	1.53 (0.60–3.90)	4.7%
	Number of mismatches	1.34 (0.56–3.21)	8.0%
	Pre-emptive transplantation	1.35 (0.53–3.39)	8.1%
	Number of transplantations	1.26 (0.48–3.28)	8.1%
	Depressive symptoms	1.25 (0.48–3.25)	8.0%

* SR: Self-reported

† EM: Electronic monitoring

## DISCUSSION

Our analysis confirmed the observation that adherence differences exist between recipients of different types of kidney graft, however, indicated living-related transplantations as the factor responsible for the lower adherence in the larger living donor transplantation group. The living-unrelated recipients showed adherence levels similar to those of deceased donor transplant recipients, which suggests that relatedness is at the core of the observed adherence differences, rather than the fact that the donor is alive and known to the recipient, as is true in most cases. This in turn agrees with our second analytic finding, *ie*, that the belief that an individual who receives a living-donor kidney needs less immunosuppressive medication than a deceased-donor kidney recipient contributed more than any other variable to the adherence difference. A sense of immunity to rejection in this group may indeed be a major factor promoting non-adherence [9]. While the explanatory power of the belief was considerable in the self-report model, its association with adherence was lower with electronic monitoring, possibly because individuals acknowledging non-adherent behavior may be more willing to admit beliefs opposing the health care provider’s. Age was also found to be an independent explanatory factor [9]. The effect of age, however, may have been underestimated in this analysis, as the clinical prototype of related donation—a young adult receiving a kidney from a parent, was uncommon in our sample.

Pre-emptive transplantation 

The seemingly large effect of pre-emptive transplantation on adherence differences observed in the Zürich is thus very likely a statistical deviation invoked by the small sample size of dialysisless transplantations in the deceased donor reference category (*ie*, n=2). Further evidence that pre-emptive transplantation does not contribute to post-transplantation adherence levels arises from the fact that living-unrelated transplant recipients (where pre-emptive transplantation is also common practice) show adherence levels similar to those of deceased donor transplants. 

A limitation of the study is that we did not have access to variables characterizing the relationship between living-(un)related donors and recipients, such as the perceived closeness or quality of the donor-recipient relationship. The emotional bond of the donor-recipient pair may be an important extra-confounding factor to be investigated.

In conclusion, our analysis supported the hypothesis of lower adherence to immunosuppressive medication in living-related kidney transplants, an association we found to be a proxy of underlying risk factors, which the modifiable risk factor “patients beliefs” (rather than experience with chronic illness) is an example of. The finding may help identifying patients at risk for rejection, which is reportedly more frequent and severe in this patient population [24], and to which non-adherent behavior has been shown to contribute.
